# What's in It for Them? A Developmental Science Perspective on Adolescent Climate Activism

**DOI:** 10.1002/jad.70187

**Published:** 2026-06-08

**Authors:** Judith van de Wetering, Katharine Lee

**Affiliations:** ^1^ Department of Psychology Utrecht University Utrecht Netherlands; ^2^ Department of Psychology University of Bath Bath UK

**Keywords:** autonomy, climate activism, connectedness, identity, motivation, social justice

## Abstract

**Introduction:**

In recent years, millions of adolescents have joined school strikes to demand climate action from governments and industries, standing in solidarity with young people from future generations and from vulnerable geographical regions (i.e., the Global South). The goal of the present study is to explore adolescents' climate activism from a developmental science perspective, analyzing how climate activism may be rooted in adolescents' developing identity and developmentally salient motives.

**Methods:**

Eleven 14–18‐year‐old Dutch adolescent climate activists (six female, two male, one non‐binary, two not disclosed) participated in an online semi‐structured interview between September 2022 and 2023. Data were analyzed in NVivo through theoretical reflexive thematic analysis, exploring patterns of meaning across the dataset while embracing researchers' active, subjective, and reflective role in data analysis.

**Results and Conclusions:**

We constructed three themes: “Activism is motivated by the desire to make contributions to a just world;” “Activism is an autonomous choice that helps explore and express who I am;” and “Activism makes me feel connected to (some but not all) others.” Taken together, the present analysis suggests that adolescents' climate activism—and pro‐environmental engagement more generally—is driven by and satisfies their developmentally salient motives to contribute to a socially just world, to make autonomous choices, to explore and express their identity, and to feel connected to others. As such, our work sheds light on how we may promote and support adolescents' engagement in acts of solidarity to contribute to today's societal challenges, and suggests avenues for further research.

## 1 Background

Adolescents growing up in the 21st century face multiple global challenges, including climate change (Crone et al. 2024; UNICEF 2023). Compared to older generations, today's adolescents will be disproportionally impacted by the consequences of climate change, including heatwaves, floods, and droughts (Thiery et al. 2021). The rapid hormonal, cognitive, and social developments during the adolescent period may especially motivate adolescents to take action for societal change (Crone et al. 2024). Indeed, adolescents have played a central role in the worldwide climate movement: in recent years, millions of adolescents have joined school strikes to demand climate action from governments and industries (de Moor et al. 2020; Wahlström et al. 2020). Adolescents' climate activism can be understood as a collective act of solidarity, particularly with young people from future generations and from vulnerable geographical regions (i.e., the Global South; Thomaes 2026). What psychological drivers may underlie adolescents' decisions to join school strikes and other forms of climate action? The current study addresses this question. Through semi‐structured interviews, we explore how climate activism may be understood by considering adolescents' developing identity and developmentally salient motivations. In so doing, we offer a novel developmental science perspective on adolescent climate activism.

## 2 Adolescence as a Window of Opportunity for Climate Activism

We define climate activism as engagement in public or collective behaviors (i.e., participation in a protest, demonstration, or action for an environmental cause, or active involvement in an environmental organization) to advocate for social or political change (Clayton and Parnes 2025). Climate activism can be understood as one form of civic engagement (defined as prosocial and political contributions to society; Buchmann 2024; Wray‐Lake and Shubert 2019) and as a manifestation of positive risk‐taking behavior (i.e., constructive behavior that entails risk; Duell and Steinberg 2019, 2021). A budding literature sheds light on young people's motivations for and perspectives on climate activism using interview and survey methods (for an overview, see Neas et al. 2022 and Romano et al. 2024). This work shows that young people are more likely to engage in climate activism when they identify with others who do so, perceive their friends to be doing so, and when they experience a sense of efficacy (Brügger et al. 2020; de Moor et al. 2020; Haugestad et al. 2021; Wahlström et al. 2020; Wallis and Loy 2021). Additionally, these studies show that young people are more likely to engage in climate activism when they are concerned about the climate crisis and feel a responsibility or even an obligation to take action (Brügger et al. 2020; Haugestad et al. 2021; Wallis and Loy 2021).

Yet this work does not typically distinguish adolescents from young adults, studying young people up to around 30 years old conjointly. Adolescence is a developmental period that starts with the onset of puberty and ends with the transition to adult social roles (Crone and Fuligni 2020; Fuligni 2019; Sawyer et al. 2018). Here, we focus on middle to late adolescence (Sawyer et al. 2018), during which prosocial behavior peaks and risk‐taking behavior sharply increases (Blankenstein et al. 2020). Adolescents may experience fewer opportunities to contribute to societal challenges like the climate crisis (e.g., not having the right to vote): For another challenge, the Covid‐19 pandemic, adolescents reported experiencing fewer opportunities to valuably contribute than young adults (te Brinke et al. 2022). Also, adolescents are not necessarily motivated to engage in climate activism in the same way as their adult counterparts.

While we acknowledge the importance of resources, skills, attitudes, and social groups for civic and activist engagement (e.g., civic voluntarism model, social identity models; Alscher et al. 2026; Buchmann 2024; Fritsche and Masson 2021; Van Zomeren et al. 2008), adolescents' motivation for climate activism may be particularly strong when they see how activist engagement is relevant to their developmentally salient motives. Although psychological motives are influential across the lifespan, they have unique manifestations during different developmental phases (Dweck 2017). Adolescence is marked by strong motives to establish autonomy, to explore identity, to feel respected by and connected to others, and to contribute to society (Branje et al. 2021; Fuligni 2019; Soenens et al. 2017; Thomaes et al. 2023; van Doeselaar et al. 2018; Yeager et al. 2018). First, adolescents expand their social worlds and seek to find their place in society (Fuligni 2019). Their social experiences accelerate the development of a range of socio‐cognitive skills (e.g., empathy, perspective‐taking, consideration for social justice; Crone et al. 2024; Hall et al. 2021). Second, hormonal changes accelerate brain developments that heighten adolescents' neurological sensitivity to their social environments and their propensity to sensation seeking (Armstrong‐Carter and Telzer 2025; Blakemore and Mills 2014; Crone and Dahl 2012; Romer et al. 2017). Together, these socio‐cognitive and neurological developments motivate adolescents to make meaningful and sometimes risky contributions to society (Blankenstein et al. 2020; Crone et al. 2024; Duell and Steinberg 2019; Fuligni 2019; Patterson et al. 2022). Indeed, the findings of one prior qualitative study focusing on adolescents' (ages 11–17) perspectives on climate activism suggested that climate activism may be relevant to adolescent motives, such as the motives to obtain respect from peers and to contribute to social justice (Lee et al. 2022). Participants in this study agreed that climate change was an issue of intergenerational injustice, and those who had taken part in strikes themselves tended to express respect for other strikers—perceiving them to be admirable and inspirational—and a belief in the potential contribution of climate strikes to climate change mitigation.

## 3 The Present Study

The goal of the present study is to explore adolescents' climate activism from a developmental science perspective, analyzing how climate activism may be rooted in adolescents' developing identity and developmentally salient motives. Our study is hypothesis‐generating, offering directions for investigating climate activism as intertwined with adolescent development and adding nuance to existing theory from adolescents' perspectives.

We expand on prior work about young people's climate activism in three ways. First, we focus on adolescence (ages 14–18) exclusively to advance insights into the motivational underpinnings of climate activism during this developmental period. Second, while most prior research is exploratory, we situate our research within a developmental science framework, hoping to advance developmentally‐attuned and holistic theorizing of adolescent climate activism (Ojala 2023). Third, we adopt an encompassing definition of climate activism that includes—but is not limited to—protesting behavior. Adolescents may contribute to climate activism in various ways, including membership of political or climate organizations and online activism. We interviewed adolescents about their activist *and* nonactivist pro‐environmental behavior, acknowledging that adolescents can adopt a variety of intertwined roles to mitigate climate change (e.g., consumers, citizens; Hampton and Whitmarsh 2023; de Ridder and Thomaes 2025).

We interviewed adolescents from The Netherlands, an affluent country in the Global North that faces comparatively low climate hazards in the near future and is also among the top 20 countries with highest CO_2_ emissions per capita (UNICEF 2021)–Dutch adolescents' climate activism may be at least in part an expression of solidarity with young people from future generations and the Global South. While Dutch adolescents are crucial stakeholders in a country that greatly impacts climate change and its mitigation, their perspectives and potential have too often been neglected.

## 4 Methods

The study was approved by the Faculty of Social and Behavioral Sciences ethics review board at Utrecht University (no 22‐0048). All study materials are accessible on Open Science Framework (https://doi.org/10.17605/OSF.IO/4FNCZ).

### 4.1 Researcher Positionality

The first author is a white, highly educated woman with a background in developmental psychology, which shaped her view of adolescence as a life stage marked by unique motivations and opportunities. The second author is a white, highly educated woman with a background in environmental social psychology. Both authors adopt a critical realist perspective, viewing a “real” world as mediated by our individual experiences and interpretations.

### 4.2 Study Design, Procedure, and Participants

We employed a qualitative cross‐sectional semi‐structured interview design. We conducted interviews because they provide in‐depth insight into complex motivational processes (Ritchie et al. 2013). While our study design does not allow us to establish causality, we refer to “motivations” and “drivers” when participants themselves framed their responses in such terms.

We recruited participants via social media, protests organized by environmental youth organizations and through snowballing. We considered 10–20 participants to be an appropriate sample size, given the study's theoretical focus, method, and practical considerations (Hennink et al. 2017). We assessed thematic saturation after data collection following Guest et al. (2020): we coded and analyzed a base size of four interviews, and then coded and analyzed two consecutive interviews to check if new (sub‐)themes emerged, until we had analyzed all interviews. No new (sub‐)themes or substantial changes in the meaning of (sub‐)themes occurred after the eighth interview.

Participants—and an appropriate caregiver if they were under 16 years old—provided written consent. They were invited for an interview if their answers to a short online survey indicated they engaged in some form of climate activism (i.e., participation in a protest, demonstration, or action for an environmental cause, or active involvement in an environmental organization). Eleven 14–18‐year‐old Dutch climate activists participated (see Table 1). Our homogenous sample matches prior descriptions of European young climate protestors as highly educated and female (de Moor et al. 2020; Wahlström et al. 2020). Importantly, except for two, participants were generally unacquainted, and they were involved in activism through different organizations (e.g., environmental and youth organizations, NGOs, government bodies, and political parties).

**TABLE 1:** Participant details.

Pseudonym	Gender	Age	Education^a^	Behaviors discussed in interview	
				Nonactivist	Activist
Emma	Female	17	Pre‐university	Taking shorter showers	Sharing photos of collected waste on social media
Anne	Female	15	Pre‐university	Shifting to a vegetarian diet	Participating in first climate protest
Sam	Rather not say	18	Pre‐university	Deciding and communicating to no longer fly for vacation	Taking on a vacant position within a youth climate organization
Jesse	Non‐binary	17	Pre‐university	Shifting to a fishless diet	Participating in first climate protest
Lisa	Female	16	Pre‐university	Shifting to a vegan diet	Participating in first climate protest
Iris	Female	15	Pre‐university	Deciding to no longer buy new clothes	Joining a youth climate conference weekend
Noah	Rather not say	16	Pre‐university	Deciding to no longer buy new clothes	Participating in a climate protest
Thomas	Male	16	Pre‐university	Deciding to no longer buy new clothes	Participating in a climate protest
Julia	Female	14	Senior general	Deciding to no longer buy new clothes	Organizing an activist theater show for a youth climate organization
Amber	Female	18	Pre‐university	Shifting to a vegan diet	Participating in a climate protest
Lucas	Male	17	Pre‐university	Shifting to a vegetarian diet	Taking on a vacant position within a green political party

^a^ All participants were in (or had just graduated from) secondary education.

The first author conducted online interviews between September 2022 and September 2023. Interviews lasted between 30 and 45 min, and were audio recorded. The semi‐structured interview schedule followed (Roulston 2010) protocol refinement framework. The interview included questions about what motivated participants to engage in climate activism, focusing on two specific moments: one in which participants engaged in nonactivist pro‐environmental behavior, and one in which they engaged in climate activist behavior (see Table 1 for behaviors discussed in the interviews). For each moment, participants were invited to describe the situation, why they chose to engage in that behavior, and to what extent they thought the behavior fit with who they were, aspired to be, or felt others thought they should be. These questions invited participants to reflect on motivational‐ and identity‐related processes that drive their behavior. Although this structure formed the basis of all interviews, the interviewer responded flexibly to each participants' responses. Participants received a €15 voucher to thank them for taking part.

### 4.3 Analysis

Interviews were transcribed verbatim. Data were analyzed in NVivo through theoretical reflexive thematic analysis (Braun and Clarke 2006, 2019), a theoretically flexible method to explore patterns of meaning across a dataset that embraces researchers' active, subjective, and reflective role in data analysis. We adopted a deductive‐inductive approach. The deductive approach took priority, given our research questions were rooted in our theoretical interest in (adolescent) motivation. However, our analysis started inductively: we generated initial codes without considering existing theory, to allow us to observe any additional patterns within the dataset. Consistent with a reflexive thematic analytical approach (Braun and Clarke 2006, 2019), we did not assess interrater reliability to establish coding consistency or consensus. Instead, we ensured quality through iterative coding, reflexive notes, co‐author and expert discussions, and the reporting of illustrative data extracts to support analytic claims, as we detail below.

The first author systematically recorded reflective notes during the study design, interviews, coding, and analysis, and revisited these regularly. Analysis proceeded through six phases, with frequent revisiting of earlier stages (Braun and Clarke 2006, 2019). First, she transcribed the interviews and noted initial thoughts and ideas. Next, she generated initial codes by systematically working through each interview. Then, she identified potential sub‐themes by shuffling and grouping printed codes. Next, she reviewed the (sub‐)themes for coherence, distinctiveness, and completeness. Then, she defined and named each theme based on analysis of its coded abstracts. Finally, the authors wrote the report, selecting relevant, illustrative quotes and linking the analysis to the research question and existing literature. At each phase, the analyses were refined and enriched through co‐author discussion and consultation with expert colleagues in (qualitative) research on adolescent pro‐environmental engagement. Throughout the Results, we indicate the number of participants (*n*) that shared a particular experience. Although frequency is not necessarily a measure of importance, it offers an indication of the extent to which a particular experience was shared across participants (Braun and Clarke 2006).

## 5 Results

**FIGURE 1:**
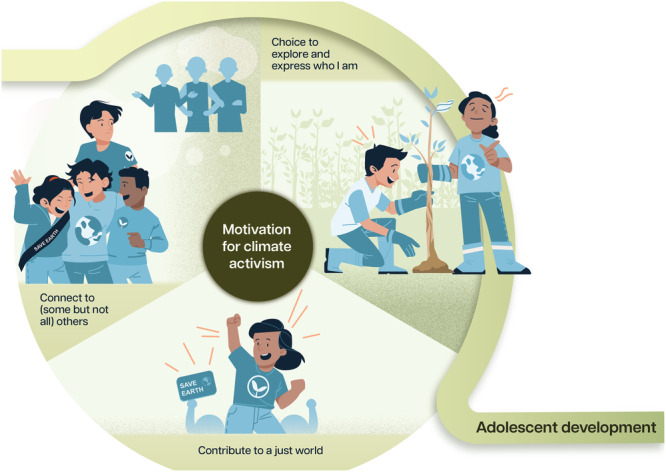
Adolescents' motivation for climate activism is intertwined with adolescent development.

We constructed three themes (see Figure 1). The first, “Activism is motivated by the desire to make contributions to a just world” explains how participants viewed their engagement as an effective and potentially impactful response to perceived injustice. The second, “Activism is an autonomous choice that helps explore and express who I am,” describes the way that participants' engagement was an independent and deliberate choice that matched, or at least partly matched, their identity. The third, “Activism makes me feel connected to (some but not all) others,” explicates the ways that participants' engagement led to an increased connection with some of their family members and peers, but also to friction with other individuals or parts of society.

### 5.1 Activism Is Motivated by the Desire to Make Contributions to a Just World

#### 5.1.1 Awareness of Injustice and Inaction

All but one participant expressed knowledge of the myriad injustices associated with climate change. For example, they described how older generations and countries in the Global North have contributed most to climate change, while younger generations and countries in the Global South will be most affected by its consequences. They also described power imbalances, for example in how humans have “ruined the planet” for other animals (Amber, 18 years), and in how polluting multinationals and their shareholders “are not the people who are also bothered by the fact that the climate is being ruined” (Iris, 15 years). At the same time, some participants (*n* = 4) were aware of the inaction of other individuals, industries, and political leaders with respect to solving the climate crisis.

#### 5.1.2 Desire to Contribute and Sense of Efficacy

These participants stated that their awareness of injustice and inaction of others made them feel motivated—sometimes even obligated—to engage in climate activism. Many (*n* = 7) characterized themselves as having “a strong concern for justice.” In this regard, participants (*n* = 6) expressed that moral emotions (e.g., anger and guilt; Pihkala 2022) and considerations (e.g., Lisa, 16 years: “Animals are living beings and in my view you don't eat living beings, that is not your food”) motivated them to engage. Through their engagement, participants wanted to make their voices heard (*n* = 3) and to contribute to a better—or at least habitable—world (*n* = 11), leaving a positive legacy for future generations (*n* = 6). Whilst one participant described (short‐lived) feelings of inefficacy as demotivating and making contributions feel “like a waste of time and energy” (Amber, 18 years), all were generally confident that their contributions were efficacious. For example, some (*n* = 6) hinted at how individual behaviors had a direct positive impact on the environment (e.g., cleaning up waste, fulfilling a vacant position in their environmental organization). Yet most participants (*n* = 9) detailed how collective behaviors (e.g., joining protests) or individual behaviors adopted at scale (e.g., switching to meatless diets or second‐hand clothing) were necessary to propel structural changes in politics, industries, and legislation, thereby indirectly but powerfully contributing to climate change mitigation.

I hear from many people that they say “Yeah, but one person doesn't make that much of a difference.” But if everyone says that… I would think: The more people become vegetarian, the less [meat] is going to be produced and, well, just especially cows, I think, cause a lot of emissions. It costs a lot of water and energy and everything.

(Anne, 15 years).

## 6 Activism Is an Autonomous Choice That Helps Explore and Express Who I Am

### 6.1 Sense of Autonomous Decision‐Making

All participants emphasized that engaging in climate activism was their personal choice, resulting from conscious deliberation. A few (*n* = 4) stressed that their choices distinguished them from the unsustainable norm. For example, Iris (15 years) described in detail how she subverted the norm by refusing to join her classmates when they went into a fast fashion store. Autonomous decision‐making was perceived to make climate activism more meaningful, as voiced by one participant.

If you just choose something yourself, then it suddenly has a lot more value to it. […] When someone chooses it for you, you actually think about it very superficially, like, just as something you have to do, not as something that's actually good or important.

(Jesse, 17 years).

### 6.2 Exploration and Expression of Identity

Some participants (*n* = 4) stated that choosing to engage in activism helped them feel authentic and coherent: “It just always feels very true to myself when I say something like that [explaining to friends that they would not fly for their vacation], because it's just really something that I want or so” (Sam, 18 years). Moreover, when asked explicitly, most participants (*n* = 10) agreed that their activism fit with their personal characteristics. They mentioned a variety of characteristics aligned with their engagement (e.g., caring, stubborn, reflective, decisive, open), suggesting that many different characteristics can be perceived as compatible with climate activism.

Nonetheless, participants did not necessarily embrace an activist identity, whether because they were not entirely comfortable with the connotations of the label (*n* = 3), because they were unsure whether they could yet claim the label (*n* = 2), or because of some concern about their level of commitment (*n* = 3). For example, Amber (18 years) described how she had struggled with her identity as a climate activist, because she realized that she had become part of a group of people whom she had always considered to be “woo woos” (i.e., irrational, crazy people). Two participants were unsure whether they identified as activists because they felt they had not been engaged enough yet to be worthy of the label: “As a person I'm very activistic, but within the climate movement I haven't done very much. I don't actually think that I can be allowed to call myself that [an activist] [but] I do intend to become one” (Iris, 15 years). Six participants wanted to distance themselves from illegal actions or did not want to invest the energy they perceived would be required.

For six participants, climate activism was a means to explore and find out how they could and wanted to contribute, by for example joining a protest to see if it was for them. These explorations sometimes engendered discomfort (*n* = 2) or were kept private (*n* = 1) because participants were unsure about whether they wanted to commit: “I'm just kind of keeping it to myself at the moment, because yeah, I'm still kind of thinking about […] how to approach things” (Jesse, 17). Thus, while all participants experienced climate activism as an autonomous choice that was—at least to some extent—compatible with their personal characteristics and identity, they expressed varying levels of current and intended future commitment to activism.

## 7 Activism Makes Me Feel Connected to (Some but not all) Others

### 7.1 Connectedness as a Driver and Consequence

All participants felt connected to others through their climate activism. They described family members as role models (*n* = 8): their discussions, enthusiasm, and lived example inspired participants to follow in their footsteps. Family also served as a support system (*n* = 7): they actively encouraged and enabled participants' engagement and voiced their support for participants' choices, even when they were also setting boundaries (e.g., not allowing their child to participate in illegal actions). Participants considered their family's support valuable, as Noah (16 years) said: “I thought it was nice to know that they [my family] wanted to join me [at the protest] and also thought it was important.” Some participants (*n* = 6) were also influenced by peers to engage in climate activism. Julia (14 years) noted: “I have a very large group of friends surrounding Fridays For Future, who all find [climate activism] very important, and I think my behavior has been very much influenced by that group of people lately.” Joining protests with peers made participants feel more at ease and the activity itself more enjoyable (*n* = 5). Through their engagement, participants (*n* = 7) also felt connected to wider social (environmental) groups that shared their interests and concerns.

### 7.2 Disconnectedness as a Caveat

However, in addition to receiving support and encouragement, all participants described prejudice and negative responses from family members, peers, or society at large towards their pro‐environmental engagement. Sam (18 years) shared: “He [My dad] sort of fills in what would be good and what would not be good for me. And in terms of sustainability, he has actually said quite often that he thinks I shouldn't lose myself in it.” More generally, Emma (17 years) realized: “If you, say, take shorter showers, eat little meat, clean up waste, for example, then people quickly think of you as some socks‐and‐sandals type of person.” Importantly, participants dealt with these conflicts in different ways. Some (*n* = 3) participants rejected people who responded negatively (e.g., actively opposing their views or deciding it was not worth it to invest energy in them), some (*n* = 5) were not bothered by opposing views (e.g., accepting differences in opinion or perceiving them as interesting), and again others (*n* = 3) struggled with the negative responses (e.g., being able to stay connected to friends who have different values from their own). One participant (Sam, 18 years) even noted that that the friction with others made them doubt whether to continue with climate activism: “I don't want to go too far, I also just want a nice life and stuff.” Thus, while participants experienced connectedness through pro‐environmental engagement, most of them also experienced disconnection from some people or parts of society.

## 8 Discussion

Recent research suggests that adolescence is a window of opportunity for solidarity, as motivational shifts and improved ability to understand others' needs and perspectives promote engagement with global challenges, such as climate change (Crone et al. 2024; Thomaes et al. 2023). Here, we interviewed eleven Dutch adolescents (ages 14–18) who engaged in climate activism about their motivation to do so. We found that adolescents' climate activism (and pro‐environmental engagement more broadly) was driven by—or at least intertwined with—developmentally salient motivations to contribute to a socially just world, to make autonomous choices, to explore and express their identity, and to feel connected to others. Thus, the current study highlights the utility of a developmental science perspective in understanding adolescents' motivation for climate activism, a manifestation of collective solidarity (de and Thomaes 2025).

### 8.1 Adolescent Climate Activism as a Contribution to a Just World

Participants exhibited high awareness of myriad injustices surrounding climate change and were motivated to contribute to a more socially just world through their climate activism. This fits with developmental science literature suggesting that adolescents' increasing capacity to understand fairness and internal motivation to be a moral person (Crone 2013; Krettenauer and Victor 2017) motivate them to contribute to society (Crone et al. 2024; Fuligni 2019; Thomaes et al. 2023). Indeed, climate justice—for example, for current and future young generations, and people in the Global South—is a central demand of youth‐led climate movements (Marris 2019; Neas et al. 2022; Thomas et al. 2019; Trott 2024). Adolescence may be an opportune developmental period for making societal contributions which, at the same time, may also help adolescents cope with the reality of climate change (Ojala et al. 2021; Sanson et al. 2019) and assist them in attaining other developmental tasks and motives (e.g., establishing autonomy, connecting with like‐minded peers; Fuligni 2019). From a developmental perspective on risk taking, climate activism may be seen as one adaptive way for adolescents to channel their propensity to take risks (e.g., when protesting, adolescents risk school reprimands and ridiculing from classmates) into behaviors that benefits society (Crone and Dahl 2012; Duell and Steinberg 2019, 2021).

Crucially, our participants generally believed that their contributions would effectively contribute to mitigation of environmental problems. This aligns with motivational frameworks for collective action that posit that individuals are motivated to engage in climate activism when they perceive that their behaviors, in combination with others', are effective (Bandura and Cherry 2020; Fritsche et al. 2018; Fritsche and Masson 2021; van Zomeren et al. 2013). It also accords with work demonstrating that adolescents with higher perceived efficacy tend to use more adaptive climate change coping strategies and engage in more pro‐environmental behavior (Ojala 2012, 2015, 2023). Contrary to a general public that typically overestimates the effectiveness of low impact climate behaviors such as recycling (Hampton and Whitmarsh 2023; IPSOS 2024), our participants were aware of individual (e.g., adopting a plant‐based diet) and collective (e.g., protesting) behaviors that can contribute to meaningful systemic change (Ivanova et al. 2020; Nielsen et al. 2021). Hence, our participants did not only experience a need to make positive societal contributions, they also experienced opportunities to effectively do so (i.e., opportunities to engage in effective pro‐environmental behaviors), something that is not always the case in youth (te Brinke et al. 2022).

### 8.2 Adolescent Climate Activism as Autonomous Choice That Is Intertwined With Identity Development

Participants spoke about their pro‐environmental engagement as a deliberate, autonomous choice, fitting with adolescents' developmentally salient motivation to act in accordance with their personal values, independently of others (Soenens et al. 2017). This motivation for autonomy can predispose adolescents to distance themselves from others (Van Petegem et al. 2015; Yeager et al. 2018)—indeed, some participants highlighted that their actions contravened unsustainable peer norms or adult expectations. Establishing autonomy is fundamental to adolescents' identity development, a process that starts in early adolescence and is characterized by exploration, a search for distinctiveness from others, and an increasing need for coherence and continuity of the self (Branje et al. 2021; van Doeselaar et al. 2018). For some participants, engaging in climate activism was a means to explore who they are and how they want to contribute. For others, their activist engagement felt deeply intertwined with their personal identity. Interestingly, participants in our study mentioned a variety of personal characteristics (e.g., being caring, stubborn, reflective, decisive, open) that aligned with their engagement. This may suggest that during adolescence, cultivating a sense of coherence (i.e., “Does this behavior fit with who I am in general?”) is particularly motivating (Thomaes et al. 2017). Crucially, participants in our study exhibited varying levels of commitment to activism, consistent with the idea that developing an identity—including an environmental (self‐)identity (Balundė et al. 2019)—is a central process during adolescence. Our observation of identity as a driver of climate activism is not new (Fritsche et al. 2018; Fritsche and Masson 2021; Vesely et al. 2021). However, the centrality of personal (rather than social) identity and of identity development (rather than content) in our adolescent sample is.

### 8.3 Adolescent Climate Activism as Motivated by Connectedness to Others

Perhaps unsurprisingly, participants attested that their climate activism was driven by the enthusiasm, lived example, and support of family members and friends. They also described feelings of connectedness resulting from climate activism, motivating them to reengage in the future. Prior work indeed identified connectedness with others as a core driver of climate activism: young people are more likely to engage in climate activism when they identify with others who do so, when they perceive that their friends and parents do so, or when they are invited to join by peers (Brügger et al. 2020; de Moor et al. 2020; Haugestad et al. 2021; Wahlström et al. 2020; Wallis and Loy 2021).

However, our participants did not only experience support and encouragement from others: their pro‐environmental engagement also caused friction. Participants described prejudice and negative responses to their climate activism from family members, peers, and society at large. Such conflicts may cause adolescents to feel alienated from some people or discouraged to engage in climate activism: While some participants said they accepted the differences between them and others, or even considered them interesting, others said they rejected people that opposed their activism or they acknowledged to find it hard to stay connected to people with different values.

### 8.4 Recommendations for Practice and Future Research

To summarize, the present study highlights how adolescent climate activism may be understood from a developmental science perspective and adds nuance to existing theory on drivers of climate activism. While requiring corroboration from larger and more diverse samples, our findings offer several directions for further investigating adolescent climate activism. One hypothesis that we derive is that that people and organizations aiming to mobilize adolescents for climate action—or similar acts of solidarity—may do well to frame such action as a means of making contributions to social justice, of establishing autonomy, of exploring who they are or aspire to be, and of connecting to others. Future longitudinal research may test whether perceived alignment between these motives and climate activism promotes adolescents' climate activism over time, and experimentally evaluate the effectiveness of motive‐aligned interventions to promote adolescents' pro‐environmental engagement (Thomaes et al. 2023; Yeager et al. 2018). Second, we hypothesize that environmental education programs—which tend to focus on promoting knowledge about climate change and effective mitigation behaviors (Świątkowski et al. 2024; van de Wetering et al. 2022)—may increase their motivational power among adolescents when they address issues of climate injustice.

Third, our work suggests that at least some adolescent climate activists may benefit from support to help them cope with negative responses from (close) others. In other words, adolescents may need to experience solidarity themselves in order to be able to act in solidarity with others. For one, adolescents may encounter peer solidarity within supportive networks that facilitate sharing experiences and building conflict resolution skills (Sanson and Bellemo 2021). Participatory research designs may involve adolescents in successfully setting up such networks (Toenders et al. 2024). Additionally, adolescent climate activists require government solidarity—while governments can facilitate adolescents' rights to peacefully protest and opportunities to express their views and influence policies, governments tend to repress protests more pre‐emptively and violently when they involve young people (UNICEF 2024).

## 9 Strengths and Limitations

By adopting a developmental psychology lens, we provide a detailed account of potential drivers of climate activism during the middle to late adolescent period. Our findings generally accord with prior work among young people (e.g., connectedness as a driver of climate activism), but offer important nuance (e.g., climate activism also produces disconnect) and directions for future work, which we hope will contribute to the formation of developmentally‐attuned theories of adolescent climate activism specifically, and collective acts of solidarity more generally. Our study centers the voices of adolescents, a demographic group that is particularly affected by and concerned about climate change and has potential to be at the forefront of much needed sustainable societal change (Hickman et al. 2021; Thiery et al. 2021; Thomaes et al. 2023).

Our study also has limitations. First, as our analysis of climate activists' motivation is based on adolescents' conscious reflections during the interviews, it is possible that our findings do not reflect motivations that adolescents were less conscious of or felt uncomfortable sharing (Custers and Aarts 2010). Also, adolescents' reflections on their motivation for climate activism must be complemented with longitudinal and experimental studies to establish directionality. Still, our qualitative approach provided rich insights into adolescents' perceived motivations. Second, our analysis of adolescent climate activists does not preclude the possibility that similar motivations underlie adults' climate activism or adolescents' nonactivist pro‐environmental behavior. Future research comparing these groups is needed to determine the extent to which specific motivational drivers (e.g., desire to make contributions, identity exploration) are indeed more central in adolescence versus adulthood, or in activist versus nonactivist behaviors. Third, this study aimed to provide an in‐depth account of adolescents' motivation to engage in climate activism from a developmental science perspective—accordingly, its sample is not generalizable. Future research should replicate and extend the present findings with larger, more diverse samples across multiple national contexts. Even though our sample reflects the broader demographic profile of young climate protesters in Europe (i.e., predominantly highly educated and female; de Moor et al. 2020; Wahlström et al. 2020), focusing on this “typical” climate activist may overlook important diversity. For instance, adolescents from marginalized groups (e.g., low‐income communities and communities of color) likely hold unique perspectives and experience unique challenges, given that climate change exacerbates existing social inequalities (Naseif et al. 2025; Neas et al. 2022; Trott 2024). Relatedly, while there is theoretical basis to assume at least some degree of similarity in the motivations for climate activism in adolescents from across the world (Dweck 2017; Thomaes et al. 2023), these motivations may manifest in different ways in different contexts (Prendergast et al. 2021). We studied the motivations of adolescent climate activists from a high‐emission country from the Global North and encourage researchers to study the motivations and experiences of adolescent climate activists in countries from the Global South, as these countries are typically the most affected by climate change while having contributed the least to its cause (UNICEF 2021). It is important to better understand how we can empower and support adolescents in such vulnerable contexts (Sanson et al. 2019).

## 10 Conclusion

What's in it for them? The present analysis suggests that adolescents' climate activism is driven by and satisfies their developmentally salient motives to contribute and to establish autonomy, identity, and connectedness. Our work highlights the potential of a developmental science lens to promote and support adolescents' engagement in acts of solidarity to contribute to global societal challenges such as climate change.

## Author Contributions

**Judith van de Wetering:** conceptualization, methodology, validation, formal analysis, investigation, data curation, writing – original draft. **Katharine Lee:** conceptualization, writing – review and editing.

## Ethics Statement

The study was approved by the Faculty of Social and Behavioral Sciences ethics review board at Utrecht University (no 22‐0048). The study was not preregistered, but the materials, final coding scheme and logs are accessible on Open Science Framework (anonymized URL: https://osf.io/4fncz/?view_only=64cc7475151842879e28aa28db98bcda).

## Conflicts of Interest

The authors declare no conflicts of interest.

## Data Availability

The data are not publicly available due to privacy constraints. Anonymized data are available from the first author upon request.
